# Heterogeneous Colorectal Cancer Risk in Women with Metabolic Dysfunction-Associated Steatotic Liver Disease by Age, Lipid, and Waist-Circumference: A Nationwide Cohort Study

**DOI:** 10.3390/cancers18010125

**Published:** 2025-12-30

**Authors:** Chang Ik Yoon, Hye Sun Lee, Soyoung Jeon, Jin Ah Lee, Dooreh Kim, Jong Min Lee

**Affiliations:** 1 Division of Breast Surgery, Department of Surgery, Seoul St Mary’s Hospital, College of Medicine, The Catholic University of Korea, Seoul 06591, Republic of Korea; fayn03@catholic.ac.kr (C.I.Y.); rlaenfpd@gmail.com (D.K.); 2 Biostatistics Collaboration Unit, Yonsei University College of Medicine, Seoul 03722, Republic of Korea; hslee1@yuhs.ac (H.S.L.); jsy0331@yuhs.ac (S.J.); 3 Department of Surgery, Yongin Severance Hospital, Yonsei University College of Medicine, Yongin 16995, Republic of Korea

**Keywords:** colorectal neoplasms, non-alcoholic fatty liver disease, women, risk factors, MASLD

## Abstract

**Background**: Metabolic dysfunction-associated steatotic liver disease (MASLD) is increasingly common and linked to obesity; however, its association with colorectal cancer (CRC) risk in women remains unclear. **Materials and Methods**: This retrospective cohort study used the Korean National Health Insurance Service health-screening database, including 483,401 women aged 40–59 years examined between 2013 and 2016, followed through 2021. MASLD was defined as hepatic steatosis (hepatic steatosis index ≥ 36 or ICD-10 K76.0) with ≥1 metabolic abnormality and no heavy alcohol use (≥20 g/day). Incident CRC (ICD-10 C18–C20) was analyzed using Cox regression adjusted for demographic, socioeconomic, and metabolic variables. Effect modification was tested across key covariates. **Results**: MASLD was found in 128,642 participants (26.6%). During a median 7.5-year follow-up, 2432 CRC cases occurred (702 with MASLD). The 7-year cumulative CRC risk was higher in the MASLD group (0.47% vs. 0.43%; *p* = 0.006). MASLD independently increased CRC risk (adjusted HR 1.10; 95% CI 1.00–1.20). Effect modification was observed for age, dyslipidemia, and waist circumference. MASLD significantly increased CRC risk among women aged 40–49 years (HR 1.26; 95% CI 1.05–1.49), those without dyslipidemia (HR 1.15; 95% CI 1.03–1.28), and with waist < 85 cm (HR 1.15; 95% CI 1.02–1.30). **Conclusions**: MASLD modestly increases CRC risk in Korean women, particularly among younger, normolipidemic, and non-obese individuals, indicating the need for age- and metabolism-specific risk stratification and suggesting a need for closer clinical attention and metabolic optimization.

## Simple Summary

Using a nationwide screening cohort of 483,401 Korean women, we found that metabolic dysfunction-associated steatotic liver disease (MASLD) is associated with an increased risk of incident colorectal cancer. Excess risk was pronounced in women aged 40–49 years, those without dyslipidemia, and those with waist < 85 cm. These findings suggest that MASLD could serve as an important marker for risk stratification, even in individuals who may appear metabolically healthy by conventional standards, thereby helping to identify women who might benefit from closer clinical attention and metabolic management.

## 1. Introduction

Metabolic dysfunction-associated steatotic liver disease (MASLD), the recently adopted term for non-alcoholic fatty liver disease (NAFLD), has emerged as a major public health issue due to its strong association with metabolic disorders such as type 2 diabetes mellitus and dyslipidemia [1]. This significant burden is evidenced by a high global prevalence ranging from 13.4% to 30.4% across various regions, while recent nationwide data in South Korea estimates the prevalence at approximately 27.5% [2,3].

Colorectal cancer (CRC) is the third most frequently diagnosed cancer and the second leading cause of cancer mortality worldwide [4], sharing several metabolic risk factors with MASLD. Recent large-scale cohort studies and meta-analyses consistently reported an increased risk of CRC in patients with NAFLD or MASLD [5,6,7]. A Korean nationwide cohort study involving over 2 million individuals showed that both NAFLD- and MASLD-based definitions were significantly associated with elevated CRC risk, with hazard ratios (HRs) ranging from 1.16 to 1.32 depending on liver fibrosis status [7].

Some evidence suggests that CRC risk may be particularly elevated in individuals with liver fibrosis, a lean phenotype, diabetes, or hypertension [6,7,8]. However, these observations remain limited and are not consistent across studies. Furthermore, data from two large European registries and a recent Swedish nationwide analysis found that excess CRC risk appeared only in men, with no statistically significant increase observed among women with NAFLD or MASLD [9,10,11].

These findings suggest that MASLD may function not only as an independent risk factor but also as a risk modifier, with heterogeneous effects across demographic and metabolic strata. In some populations—particularly women—MASLD’s impact on CRC risk may be negligible. Clarifying whether MASLD confers excess CRC risk in women, and identifying which subgroups are disproportionately affected, is essential for improving risk stratification and guiding targeted preventive strategies.

Therefore, we used a nationwide, population-based female cohort to (i) quantify the overall association between MASLD and incident CRC and (ii) explore effect modification across predefined subgroups to clarify whether MASLD constitutes a CRC risk factor in women.

## 2. Materials and Methods

### 2.1. Database

This study established a nationwide retrospective cohort using integrated data provided by the Health and Medical Big Data Integration Platform, which consolidates records from the National Health Insurance Service (NHIS), Health Insurance Review and Assessment Service (HIRA), Korea Central Cancer Registry (KCCR), and Statistics Korea. Access to this population-level data was granted by the institutional ethics committee (local IRB number: KC23ZISI0410, 15 June 2023) and the governmental data access committee (Project No. 2023-00025). The database covers nearly the entire Korean population and provides longitudinal information on demographics, health-screening records, socioeconomic status, medical diagnoses, prescriptions, cancer registrations, and verified causes of death. However, owing to the limitations associated with large-scale data extraction, approximately one million women aged 40–59 years were randomly sampled by region from the overall eligible population. All personal identifiers were removed prior to the analysis. Given the retrospective nature of this study, the requirement for written informed consent was waived and was conducted in accordance with the applicable data protection regulations and ethical standards of the Republic of Korea.

### 2.2. Study Design and Enrollment Criteria

The study population consisted of women aged 40–59 years who underwent at least one national general health screening between 1 January 2013, and 31 December 2016. The date of the first screening was defined as the index date. As the NHIS database records only the year of examination and not the exact date, the year of the first screening was considered the year of cohort entry. The preceding year served as a washout period.

Follow-up for incident CRC (International Classification of Diseases, 10th Revision [ICD-10] codes: C18–C20) began on January 1 of the year after cohort entry and continued until 31 December 2021. The specific inclusion and exclusion criteria for study enrollment are summarized in Table 1. Diagnostic codes used for the exclusion criteria are listed in Supplementary Table S1.

### 2.3. Definition of Variables

Baseline demographic, clinical, socioeconomic, and lifestyle characteristics were obtained from NHIS records. Physical activity was assessed from responses to a health-screening questionnaire, which included the number of days participants engaged in walking, moderate-intensity physical activity, and vigorous-intensity physical activity per week. The total weekly physical activity score was calculated based on the estimated metabolic equivalent of task (MET) values assigned to each activity level. These scores were then categorized into quartiles to define physical activity levels for analysis.

Comorbidities, including hypertension, diabetes mellitus, dyslipidemia, and cardiovascular disease, were identified using ICD-10 codes [12] from inpatient and outpatient claims (Supplementary Table S1). To assess the overall burden of comorbidities, we used the Charlson Comorbidity Index (CCI) [13], a validated scoring system widely used to quantify the severity of chronic conditions. Each comorbidity category was assigned a weighted score, and the total CCI score was included in the multivariable analyses to adjust for the potential confounding effects of chronic diseases (Supplementary Table S2).

### 2.4. Assessment of MASLD

MASLD was defined according to the latest international criteria and included hepatic steatosis, metabolic dysfunction, and alcohol consumption. Participants were classified as having MASLD if they had a documented diagnosis of fatty liver (ICD-10 code: K76.0) or met the specific criteria based on the Hepatic Steatosis Index (HSI). The detailed components and thresholds used for the MASLD definition are summarized in Table 2.

The HSI is a widely validated prediction model that incorporates the aspartate transaminase/alanine transaminase ratio, BMI, sex, and the presence of diabetes mellitus. The HSI has shown good predictive performance for fatty liver in the Korean population, with an area under the receiver operating characteristic curve of 0.82 (95% confidence interval [CI] 0.81–0.83) at values above 36 [14].

Although the FLI, similar to the HSI, is a useful quantitative tool for predicting the presence of MASLD without imaging studies or biopsies [15], its reliance on a greater number of variables, such as waist circumference, triglycerides, and gamma-glutamyl transferase levels, led us to use only the HSI to identify the MASLD group in our study.

### 2.5. Study Outcomes

The primary outcome was incident CRC. In addition, the potential for effect modification was evaluated across pre-specified subgroups, including age, dyslipidemia, waist circumference, BMI category, smoking status, physical activity, alcohol intake, and comorbidity burden.

### 2.6. Statistical Analyses

Baseline demographic and clinical characteristics were compared using Student’s t-test for continuous variables and the chi-squared test for categorical variables. The cumulative incidence of CRC was estimated using Kaplan–Meier curves and compared using the log-rank test. Univariate and multivariate Cox proportional hazards regression models were used to evaluate factors associated with the outcomes. The proportional hazards assumption was verified using log-log survival plots. The analysis was conducted using a complete-case approach, including only participants with no missing values for the key covariates. A multivariate model was built using the stepwise method and adjusted for relevant covariates. Statistical significance was defined as two-sided *p* < 0.05. Effect modification was evaluated using cross-product interaction terms in the Cox model. Interactions were considered suggestive at *p* for interaction < 0.15, and those with opposite directions of association between strata were interpreted as indicative of effect modification [16]. All statistical analyses were performed using the SAS software (version 9.3; SAS Institute Inc., Cary, NC, USA).

## 3. Results

### 3.1. Baseline Characteristics

**Figure 1:**
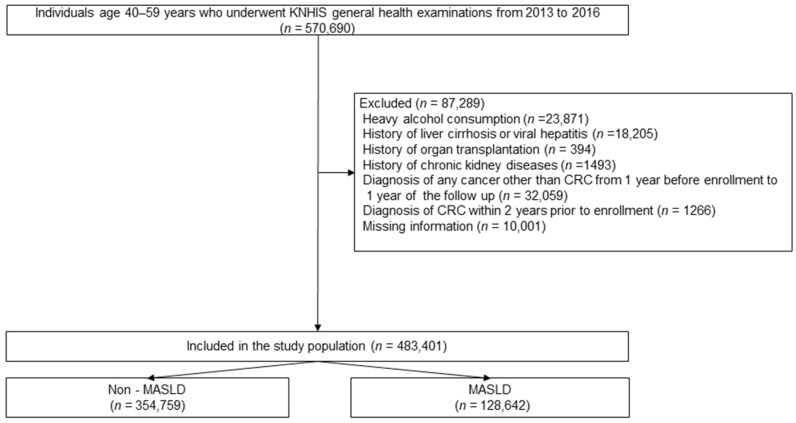
Study population selection flowchart. KNHIS, Korean National Health Insurance Service; CRC, colorectal cancer; MASLD, metabolic dysfunction-associated steatotic liver disease.

From an initial database of 570,690 women aged 40–59 years who underwent the National Health Screening between 2013 and 2016, 87,289 were excluded due to duplicate records, missing key variables, heavy alcohol use (≥20 g/day), viral hepatitis or cirrhosis, organ transplantation, or a prior cancer diagnosis. The final study cohort included 483,401 participants, of whom 128,642 (26.6%) had MASLD, and 354,759 (73.4%) did not (Figure 1).

Compared with the non-MASLD group, women in the MASLD group were more likely to be aged 50–59 years (69.8% vs. 62.7%; *p* < 0.001), severely obese (BMI ≥ 30 kg/m^2^: 25.2% vs. 0.1%; *p* < 0.001), and centrally obesity (waist circumference ≥ 85 cm: 55.3% vs. 7.8%; *p* < 0.001). They were also more likely to report low physical activity (lowest quartile: 27.7% vs. 22.9%; *p* < 0.001) and to be in the lower income strata (32.0% vs. 34.7%; *p* < 0.001). The MASLD group also had higher rates of diabetes mellitus (22.3% vs. 6.1%), hypertension (37.2% vs. 17.0%), dyslipidemia (35.0% vs. 22.2%), and cardiac disease (14.0% vs. 9.0%) (all *p* < 0.001), resulting in a higher mean CCI (3.18 ± 1.40 vs. 2.69 ± 1.04; *p* < 0.001) (Table 3).

### 3.2. Factors Associated with Incident CRC

**Figure 2:**
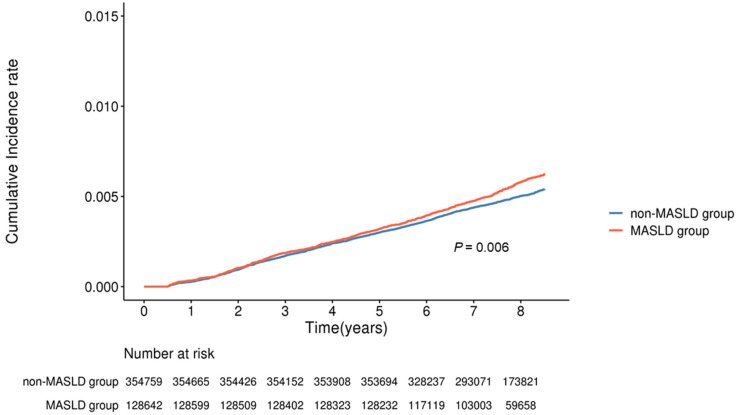
Kaplan–Meier curves showing the cumulative incidence of CRC in the MASLD and non-MASLD groups.

The median follow-up was 7.51 years (interquartile range, 7.51–8.51) in both groups. During this period, 2432 incident CRC cases were identified: 702/128,642 (0.55%) in the MASLD group and 1730/354,759 (0.49%) in the non-MASLD group. Kaplan–Meier analysis demonstrated a significantly higher cumulative incidence of CRC in the MASLD group than that in the non-MASLD group (Figure 2, log-rank *p* = 0.006). The estimated cumulative risks of CRC at 3, 5, and 7 years were 0.18% vs. 0.17%, 0.32% vs. 0.30%, and 0.47% vs. 0.43% in the MASLD and non-MASLD groups, respectively. In the multivariate Cox proportional hazards model (Table 4), the following variables were independently associated with CRC: age 50–59 years (HR 1.508, 95% CI 1.378–1.650), *p* < 0.001), highest income decile (8–10) (HR 0.755, 95% CI 0.579–0.986, *p* = 0.038), current smoker (HR 1.329, 95% CI 1.068–1.653, *p* = 0.01), and MASLD (HR 1.095, 95% CI 1.003–1.195, *p* = 0.044).

### 3.3. Subgroup Analyses

**Figure 3:**
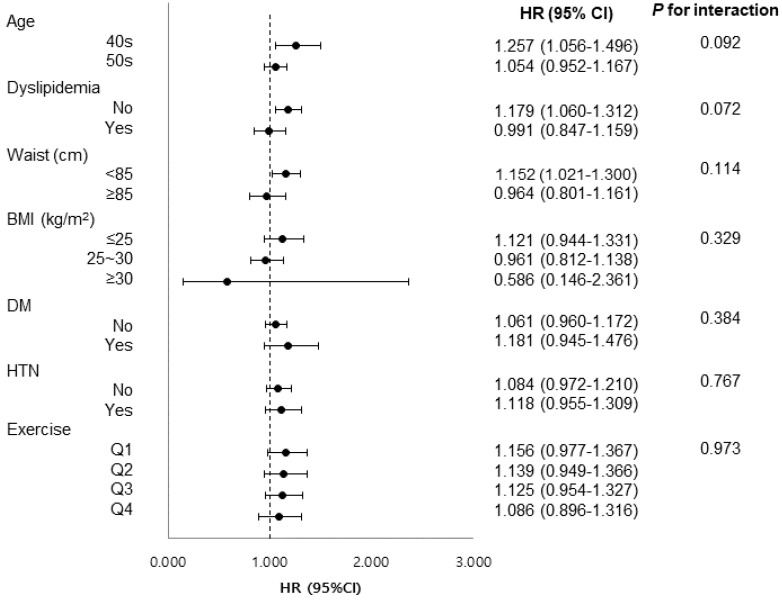
Subgroup analysis of the association of MASLD with colorectal cancer risk. HR, hazard ratio; CI, confidence interval; BMI, body mass index; DM, diabetes mellitus; HTN, hypertension.

As shown in Figure 3, an effect modification was detected for age, dyslipidemia status, and waist circumference. In the stratified multivariate models, MASLD was independently associated with CRC risk among women aged 40–49 years (HR 1.261, 95% CI 1.049–1.501, *p* = 0.009), those without dyslipidemia (HR 1.146, 95% CI 1.030–1.276, *p* = 0.012), and those with waist circumferences < 85 cm (HR 1.151, 95% CI 1.020–1.298, *p* = 0.022). In the complementary strata (age 50–59 years, presence of dyslipidemia, or waist circumference ≥ 85 cm), MASLD was not significantly associated with CRC risk.

## 4. Discussion

This nationwide analysis of 483,401 Korean women showed that MASLD was independently associated with a significant increase in incident CRC, even after adjusting for demographic, socioeconomic, and metabolic covariates. Importantly, this excess hazard was not uniform; it was concentrated in women aged 40–49 years, those without clinically coded dyslipidemia, and those with a waist circumference of <85 cm. These findings extend current knowledge by demonstrating the heterogeneous nature of MASLD’s oncogenic impact; while the associated risk is modest, it may suggest meaningful public health implications when considered on a nationwide scale.

Previous studies reported mixed conclusions regarding the association between MASLD (or NAFLD) and CRC in women. In the Swedish National Patient Registry [10], which included approximately 80,000 individuals with NAFLD, the HR for CRC was 1.54 (95% CI 1.13–2.08) in men but only 1.21 (0.84–1.73) in women. Similarly, a Korean hospital screening cohort of 25,947 participants showed a similar sex contrast, with HRs of 2.21 (1.26–3.87) for men and 1.00 (0.37–2.70) for women [9]. By contrast, a Korean national screening study [7] of 8.9 million adults reported significant associations in both sexes, implying that null results in smaller cohorts of women may reflect limited statistical power.

Apart from the differences in case numbers between studies, several methodological and biological factors may explain these discrepant findings. First, nearly two-thirds of women in our cohort were aged ≥ 50 years and therefore peri- or post-menopausal, whereas many earlier cohorts of women were predominantly premenopausal. Estrogen deficiency accelerates visceral fat accumulation, hepatic insulin resistance, and systemic inflammation, all of which promote colorectal carcinogenesis [17,18]. The older hormonal profile in our cohort may therefore provide a plausible biological setting in which the MASLD-CRC association becomes detectable, whereas younger estrogen-replete populations may mask this effect. Second, our study applied a stricter definition of MASLD than that used in earlier women-null reports. We required hepatic steatosis, indicated by an HSI of ≥36, in combination with at least one metabolic abnormality, whereas previous studies often relied solely on ultrasound findings or registry codes to define NAFLD [9,10]. This stricter definition reduces the inclusion of metabolically benign steatosis and enriches for women in whom hepatic fat coexists with systemic metabolic stress. Such enrichment aligns more closely with the biological pathways that promote colorectal tumorigenesis, providing further rationale for the positive association observed in our data.

Importantly, the association between MASLD and CRC differed according to age. In our cohort, women aged 40–49 years had an HR of 1.254 (95% CI 1.054–1.493), whereas those aged 50–59 years showed no significant increase (1.047 [0.946–1.159]). Similarly, a prospective Chinese cohort of 63,696 adults found that NAFLD diagnosed before the age of 45 years carried the greatest digestive cancer risk, with the hazard attenuating at older ages. Early-onset MASLD may lead to prolonged hepatic lipotoxicity and chronic subclinical inflammation, thereby providing a longer window for malignant transformation. Mechanistic studies of early-onset CRC (EOCRC) strengthen this interpretation [19]. Population data from the United States and Europe have shown a rapid increase in EOCRC, which parallels increasing obesity and metabolic dysfunction in younger adults. Previous studies have linked hyperinsulinemia and activation of the insulin-like growth factor axis to enhanced proliferation and reduced apoptosis of colonic epithelial cells [20]. Recent evidence indicated that NAFLD is independently associated with a higher risk of EOCRC, particularly in the left colon and rectum [21].

In MASLD, excessive production of secondary bile acids (BAs), accumulation of deoxycholic acid, and gut microbial dysbiosis can promote intestinal inflammation and carcinogenesis [22]. However, statins, which are commonly prescribed to patients with dyslipidemia, inhibit HMG-CoA reductase, thereby lowering the production of secondary BAs and reducing the intestinal BA load [23]. This modulation may attenuate β-catenin activation and oxidative DNA damage in the colon. Large meta-analyses have shown that statin therapy, routinely prescribed to most individuals with ICD-10 code E78 (dyslipidemia), reduces CRC incidence by approximately 10% [24]. This mechanism could partly explain why the carcinogenic effect of MASLD was not evident in dyslipidemic women but became apparent in those without dyslipidemia. Furthermore, women classified as normolipidemic are generally untreated and may carry atherogenic remnant-rich lipoproteins that are not captured by standard lipid panels [25]. These findings imply that pharmacological lipid-lowering therapy can mask the MASLD-linked CRC risk, whereas residual metabolic dyslipidemia may expose this risk.

Finally, MASLD predicted CRC only among women with a waist circumference < 85 cm. While visceral obesity is a well-established driver of CRC, many studies have shown that NAFLD in lean or non-obese individuals confers a stronger CRC risk than NAFLD in their obese counterparts [26,27]. Lean NAFLD patients often exhibit insulin resistance and dyslipidemia, and these metabolic abnormalities may promote colorectal carcinogenesis through activation of the insulin–IGF signaling pathway [28]. However, it remains difficult to clearly explain why the MASLD–CRC association was not observed in obese women. One possible explanation is that in women with central obesity, the baseline CRC risk is already high, leaving little additional risk attributable to hepatic fat.

Our findings imply that MASLD status may serve as an additional marker for risk stratification, particularly in women aged 40–59 years. First, incorporating simple steatosis indices into routine assessments could assist in identifying women with potential metabolic risks. This would facilitate the identification of women with occult metabolic risks who might otherwise be overlooked, such as those with a normal BMI or waist circumference but significant hepatic steatosis. Implementing these indices as a primary screening tool could enhance the “triage” process for CRC surveillance in primary care settings. Second, while Korean screening guidelines are lowering the initiation age—from 50 in the national program to 45 in academic recommendations—to address the rising incidence of EOCRC, metabolic risk assessment is not yet integrated. Diversifying the screening initiation age or customizing screening intensity through risk stratification based on metabolic markers, such as MASLD, could potentially enhance early detection and optimize resource allocation. Third, clinicians should continue to emphasize metabolic optimization—including lifestyle modification and management of dyslipidemia—which may help mitigate CRC risk in MASLD-positive women who appear otherwise metabolically healthy. Recognizing these associations could contribute to a more comprehensive approach to CRC prevention in this specific population.

Future research should address not only the biological mechanisms linking MASLD to colorectal carcinogenesis but also conduct studies in diverse cohorts to reach a consensus on the heterogeneity of CRC risk across subgroups. Furthermore, the cumulative impact of MASLD duration and severity is worth exploring. Such evidence will be essential to establishing precise risk stratification models and enhancing our understanding of MASLD-associated carcinogenesis.

This study had several limitations that warrant consideration. First, its retrospective, claims-based design precludes causal inference and allows for residual or unmeasured confounding factors despite extensive covariate adjustment. Second, key exposures, including alcohol intake, physical activity, and medication use, were captured only at baseline; changes during follow-up could not be modeled, potentially leading to misclassification. Third, this cohort comprised Korean women aged 40–59 years who participated in the national screening program; therefore, the findings may not be generalizable to men, other ethnicities, or younger and older age groups. Fourth, we lacked data on menopausal hormone therapy, dietary patterns, and gut microbiome composition, which prevented the assessment of their potential effect-modifying roles. Finally, we were unable to account for competing risks of non-CRC mortality, which might have led to an overestimation of the cumulative incidence of CRC. These limitations should be weighed against the strengths of our large nationally representative cohort when interpreting the clinical implications of MASLD-associated CRC risk in women.

## 5. Conclusions

In conclusion, in this large nationwide cohort of Korean women, MASLD was independently associated with a 10% increase in incident CRC. Excess risk was confined to women aged 40–49 years, those without dyslipidemia, and those with a waist circumference of <85 cm, underscoring the substantial heterogeneity in MASLD-related carcinogenesis. These findings highlight the importance of age- and metabolism-specific risk stratification, suggesting that MASLD status could serve as a marker to identify women who may benefit from closer clinical attention and metabolic optimization.

## Figures and Tables

**Table 1:** Study enrollment and selection criteria.

Data Sources	Inclusion Criteria	Exclusion Criteria
Nationwide cohort (NHIS, HIRA, KCCR, Statistics of Korea) of women who participated in the National Health Screening Program between 2013 and 2016.	Aged 40–59 years at the time of the initial health screening during the study period (2013–2016)	History of colorectal cancer (ICD-10: C18–C20) within two years prior to the baseline health screening date
		Diagnosis of any cancer other than colorectal cancer (ICD-10: C00–C97, excluding C18–C20) within one year before or after the baseline health screening date.
		Heavy alcohol consumption (≥20 g/day).
		Liver cirrhosis, viral hepatitis, chronic kidney disease, or organ transplantation.
		Missing data on key covariates *.

Abbreviations: HIRA, Health Insurance Review and Assessment Service; KCCR, Korea Central Cancer Registry; NHIS, National Health Insurance Service. * Key covariates were defined as household income, smoking status, alcohol consumption, physical activity, and metabolic parameters required for the definition of MASLD.

**Table 2:** Definition of MASLD.

MASLD Was Defined If All Three of the Following Criteria Were Met:		
Hepatic steatosis	Metabolic dysfunction	Alcohol consumption
HSI ≥ 36	Presence of at least one of the following five metabolic risk factors:	Non-heavy alcohol intake (<20 g/day).
	- BMI ≥ 23 kg/m^2^ or waist circumference ≥ 85 cm	
	- fasting plasma glucose level of ≥100 mg/dL, antidiabetic medication use, or a diagnosis of type diabetes mellitus (ICD-10 code: E11)	
	- systolic blood pressure ≥ 130 mmHg, diastolic blood pressure ≥ 85 mmHg, or antihypertensive medication use	
	- triglyceride levels ≥ 150 mg/dL or lipid-lowering medication use	
	- HDL cholesterol < 50 mg/dL or lipid-lowering medication use	

Abbreviations: BMI, body mass index; HDL, high-density lipoprotein; HSI, hepatic steatosis index; ICD-10, International Classification of Diseases, 10th Revision; MASLD, metabolic dysfunction-associated steatotic liver disease. Note: HSI was calculated using the following formula: 8 × (ALT/AST) + BMI + 2 (female) + 2 (if diabetic).

**Table 3:** Baseline characteristics of the study population.

	Non-MASLD (*n* = 354,759)	MASLD (*n* = 128,642)	*p*-Value
Age, years, No. (%)			<0.001
40–49	132,168 (37.3)	38,835 (30.2)	
50–59	222,591 (62.7)	89,807 (69.8)	
BMI, kg/m^2^, No. (%)			<0.001
≤25	317,601 (89.5)	26,633 (20.7)	
25–30	36,956 (10.4)	69,633 (54.1)	
≥30	202 (0.1)	32,376 (25.2)	
Waist circumference, cm, No. (%)			<0.001
<85	326,996 (92.2)	57,454 (44.7)	
≥85	27,763 (7.8)	71,188 (55.3)	
Income deciles *, No. (%)			<0.001
0	6381 (1.8)	3475 (2.7)	
1–3	105,785 (29.8)	37,965 (29.5)	
4–7	119,526 (33.7)	46,102 (35.8)	
8–10	123,067 (34.7)	41,100 (32.0)	
Smoking status, No. (%)			<0.001
Never	340,887 (96.1)	123,210 (95.8)	
Former	4525 (1.3)	1829 (1.4)	
Current	9347 (2.6)	3603 (2.8)	
Weekly alcohol consumption, No. (%)			<0.001
None	266,993 (75.3)	102,400 (79.6)	
1–2	78,837 (22.2)	24,040 (18.7)	
3–4	8016 (2.3)	1961 (1.5)	
5–7	913 (0.3)	241(0.2)	
Physical activity quartile ^†^, No. (%)			<0.001
Q1	81,205 (22.9)	35,625 (27.7)	
Q2	84,705 (24.0)	31,215 (24.3)	
Q3	101,106 (28.5)	35,208 (27.4)	
Q4	87,743 (24.7)	26,594 (20.7)	
Diabetes mellitus, No. (%)	21,646 (6.1)	28,738 (22.3)	<0.001
Hypertension, No. (%)	60,367 (17.0)	47,845 (37.2)	<0.001
Dyslipidemia, No. (%)	78,699 (22.2)	45,076 (35.0)	<0.001
Cardiac disease, No. (%)	31,940 (9.0)	17,980 (14.0)	<0.001
CCI, mean ± SD	2.69 ± 1.04	3.18 ± 1.40	<0.001

Abbreviations: MASLD, metabolic dysfunction-associated steatotic liver disease; No., number; BMI, body mass index; CCI, Charlson comorbidity index; SD, standard deviation. * Income decile grouped as: 0 (lowest), 1–3 (low), 4–7 (middle), 8–10 (high). ^†^ Q1–Q4 indicate increasing levels of physical activity, from lowest (Q1) to highest (Q4).

**Table 4:** Cox proportional hazards regression of risk factors for incident colorectal cancer.

	Univariable		Multivariable *	
	HR (95% CI)	*p*-Value	HR (95% CI)	*p*-Value
MASLD	1.130 (1.035–1.234)	0.006	1.095 (1.003–1.195)	0.044
Age, years				
40–49	ref		ref	
50–59	1.510 (1.380–1.652)	<0.001	1.508 (1.378–1.650)	<0.001
BMI, kg/m^2^				
≤25	ref			
25–30	1.143 (1.040–1.256)	0.005		
≥30	1.206 (1.036–1.403)	0.015		
Waist circumference, cm				
<85	ref			
≥85	1.128 (1.026–1.241)	0.001		
Income deciles				
0	ref		ref	
1–3	0.873 (0.670–1.137)	0.313	0.884 (0.677–1.152)	0.361
4–7	0.861 (0.661–1.121)	0.266	0.857 (0.658–1.117)	0.254
8–10	0.752 (0.577–0.980)	0.034	0.755 (0.579–0.986)	0.038
Smoking status				
Never	ref		ref	
Former	0.848 (0.576–1.248)	0.401	0.874 (0.594–1.287)	0.495
Current	1.337 (1.075–1.662)	0.009	1.329 (1.068–1.653)	0.010
Weekly alcohol consumption				
None	ref			
1–2	0.964 (0.873–1.063)	0.461		
3–4	0.821 (0.603–1.119)	0.212		
5–7	1.388 (0.694–2.778)	0.353		
Physical activity quartile				
Q1	ref			
Q2	0.928 (0.827–1.040)	0.198		
Q3	0.985 (0.884–1.098)	0.788		
Q4	0.935 (0.833–1.048)	0.248		
Diabetes mellitus	1.328 (1.181–1.493)	<0.001		
Hypertension	1.189 (1.086–1.303)	<0.001		
Dyslipidemia	1.127 (1.031–1.231)	0.008		
CCI	1.031 (0.997–1.065)	0.073		

Abbreviations: HR, hazard ratio; CI, confidence interval; MASLD, metabolic dysfunction-associated steatotic liver disease; BMI, body mass index; CCI, Charlson Comorbidity Index. * Multivariable Cox model was constructed using a stepwise variable selection procedure that included all candidate covariates; only variables retained in the final model are shown.

## Data Availability

The underlying NHIS data are stored in a secure research environment and cannot be shared publicly. Access is limited to approved researchers through the NHIS Data Access Committee.

## Supplementary Materials

The following supporting information can be downloaded at https://www.mdpi.com/article/10.3390/cancers18010125/s1, Table S1: Diagnostic codes; Table S2: Charlson Comorbidity Index.
