# Supplementary material for: Heterogeneous Colorectal Cancer Risk in Women with Metabolic Dysfunction-Associated Steatotic Liver Disease by Age, Lipid, and Waist-Circumference: A Nationwide Cohort Study
Source: Cancers (Basel). 2025 Dec 30;18(1):125. doi: 10.3390/cancers18010125 (PMC12784733; doi:10.3390/cancers18010125)
Supplement: Supplementary file 1 [file cancers-18-00125-s001.zip › cancers-4036591-supplementary.pdf]

Table S1. Diagnostic codes.

Classification	Diagnosis	ICD-10 code
Liver cirrhosis	Liver cirrhosis	K70.3
Viral hepatitis	Viral hepatitis	B15, B16, B17, B18, B19
Renal failure	Renal failure	N18
Hypertension	Hypertension	I10, I11.0, I11.9, I12.0, I12.9, I13.0, I13.2, I13.11, I15, N26.2
Dyslipidemia	Dyslipidemia	E78
Cardiac disease	Acute myocardial infarction	I21, I22, I23
	Heart failure	I11.0, I13.0, I13.2, I42.0, I42.1, I42.3, I42.4, I42.5, I42.6, I42.7, I42.8, I42.9, I43, I50.0, I50.1, I50.20, I50.21, I50.22, I50.23, I50.30, I50.31, I50.32, I50.33, I50.40, I50.41, I50.42, I50.43, I50.9, R57.0
	Ischemic stroke	I63, I65, I66, I67.84, I67.89, G45.0, G45.1, G45.2, G45.8, G45.9, G46.0, G46.1, G46.2
	Coronary artery disease	I20, I21, I22, I24, I25
	Peripheral artery disease	I70.2-I70.9, I71, I73.9, I74.2, I74.3, I74.4, I74.5, I77.89, I77.9
	Valve disorder	I05, I06, I07, I08, I09, I34-I37
	Atrial fibrillation	I48
Transplantation	Kidney transplant status	Z94.0
	Heart transplant status	Z94.1
	Lung transplant status	Z94.2
	Heart and lungs transplant status	Z94.3
	Liver transplant status	Z94.4
	Skin transplant status	Z94.5
	Bone transplant status	Z94.6
	Corneal transplant status	Z94.7
	Other transplanted organ and tissue stats	Z94.8, Z94.89
	Bone marrow transplant status	Z94.81
	Intestine transplant status	Z94.82
	Pancreas transplant status	Z94.83
	Stem cells transplant status	Z94.84
	Transplanted organ and tissue status, unspecified	Z94.9

Table S2. Charlson Comorbidity Index.

Comorbidity	ICD-10 code	Updated Weight
Myocardial infarction	I21.x, I22.x, I25.2	0
Congestive heart failure	I09.9, I11.0, I13.0, I13.2, I25.5, I42.0, I42.5-I42.9, I43.x, I50.x, P29.0	2
Peripheral vascular disease	I70.x, I71.x, I73.1, I73.8, I73.9, I77.1, I79.0, I79.2, K55.1, K55.8, K55.9, Z95.8, Z95.9	0
Cerebrovascular disease	G45.x, G46.x, H34.0, I60.x, I69.x	0
Dementia	F00.x-F03.x, F05.1, G30.x, G31.1	2
Chronic pulmonary disease	I27.8, I27.9, J40.x-J47.x, J60.x-J67.x, J68.4, J70.1, J70.3	1
Rheumatologic disease	M05.x, M06.x, M31.5, M32.x, M34.x, M35.1, M35.3, M36.0	1
Peptic ulcer disease	K25.x-K28.x	0
Mild liver disease	B18.x, K70.0-K70.3, K70.9, K71.3-71.5, K71.7, K73.x, K74.x, K76.0, K76.2-K76.4, K76.8, K76.9, KZ94.4	2
Diabetes without chronic complication	E10.0, E10.1, E10.6, E10.8, E10.9, E11.0, E11.1, E11.6, E11.8, E11.9, E12.0, E12.1, E12.6, E12.8, E12.9, E13.0, E13.1, E13.6, E13.8, E13.9, E14.0, E14.1, E14.8, E14.9	2
Diabetes with chronic complication	E10.2-E10.5, E10.7, E11.2-E11.5, E11.7, E12.2-E12.5, E12.7, E13.2-E13.5, E13.7, E14.2-E14.5, E14.7	1
Hemiplegia or paraplegia	G04.1, G11.4, G80.1, G80.2, G81.x, G82.x, G83.0-G83.4, G83.9	2
Renal disease	I12.0, I13.1, N03.2-N03.7, N05.2-N05.7, N18.x, N19.x, N25.0, Z49.0-Z49..2, Z94.0, Z99.2	1
Any malignancy including leukemia and lymphoma	There are no eligible patients in this study	2
Moderate or severe liver disease	I185.0, I85.9, I86.4, I98.2, K70.4, K71.1, K72.1, K72.9, K76.5, K76.6, K76.7	4
Metastatic solid tumor	There are no eligible patients in this study	6
Acquired immune deficiency syndrome/human immunedeficiency virus	B20.x-B22.x, B24.x	4
